# Bedtime Story to My Mother: Virgin Females Seek Love †

**DOI:** 10.3390/insects17020146

**Published:** 2026-01-27

**Authors:** Marc Rhainds

**Affiliations:** Atlantic Forestry Centre, Canadian Forest Service, Natural Resources Canada, P.O. Box 4000, Fredericton, NB E3B 5P7, Canada; marc.rhainds@nrcan-rncan.gc.ca

**Keywords:** bagworms, female mating effort, mass trapping, mating disruption, pheromone-calling behavior, Psychidae, reproductive isolation, spruce budworm

## Abstract

The probability that female bagworms (Lepidoptera: Psychidae) are in mating time-in (live pheromone calling) was recorded in three bagworm species: *Oiketicus kirbyi* in a Costa Rican oil palm plantation in 1993–1994; *Metisa plana* in Malaysian oil palm plantations during five consecutive generations of bagworms in 1996; and *Thyridopteryx ephemeraeformis* on ornamental trees in the Midwest United States. Because females entirely reproduce within their bag (mate attraction, copulation, and oviposition), it is possible to assess the mating success of time-out females (dead individuals from an ongoing generation that either mated or died as a lifelong virgin) and incidence of calling females that may or may not mate before death. Synchronous larval development and discrete (non-overlapping) generations imply a declining proportion of live calling females over time in all three bagworm species: ‘young’ calling females prevail in the early season as opposed to a majority of time-out (post-reproductive) females in the late season. Calling females are long-lived relative to males (one-day lifespan) and thus expected to mate as adults when abundance of males is high and/or female longevity exceeds three days. A low mating success of calling females is associated with extreme protogyny (early season male shortage; *O. kirbyi* in 1994) or late adult emergence in populations at the edge of the distribution range (*T. ephemeraeformis* at latitudes > 41° N in 2019).

## Simple Summary

In organisms with obligatory sexual reproduction, females are born virgin and need to access male sperm over the course of their life to produce offspring. This problem is simplified in holometabolous insects with complete metamorphosis because food acquisition and sexual activities are segregated into separate life history phases (transition between larval, pupal, and adult stages). Unfortunately, female transition from virgin to mated status in feral populations is challenging to quantify due to the high mobility, small size, and cryptic nature of adult insects. Neotenic female bagworms reproduce within a self-constructed bag, thus providing a model system to parameterize reproductive processes.

## 1. Introduction

Sexual communication in moths is usually mediated by a female-emitted volatile signal (sex pheromone) for the attraction of males [1,2,3]. Calling females actively releasing pheromone for mate attraction are deemed to be in a mating time-in phase, as opposed to non-calling females who are in a time-out phase [4,5,6,7,8,9]. At the intraspecific level, duration of mating time-in (signalling effort) is amplified for old virgins that call earlier at night and for a longer period than young virgins due to increased risk that females do not mate as adults (matelessness/mating failure/lifelong virginity/zerogamy) [1,10,11,12,13,14].

Polyandry (females mate more than once) implies sequential shifts between mating time-in/time-out during life [15,16]. This problem is simplified in flightless monandrous females that enter a permanent mating time-out phase after successful copulation [17,18,19,20]. With that said, measuring diurnal variation in pheromone emission in large groups of feral females (‘true time-in’: pulsed release of pheromone) is often impractical. In addition, sampling constraints vastly challenge assessment of mating status in feral populations of females owing to the small size/cryptic nature of insects [21,22,23]. The problem is compounded in phenology studies that require repeated observations of mating time-in during the entire emergence season.

The life history of bagworms (Lepidoptera: Psychidae) provides a model system to investigate the incidence of mating time-in due to the unusual tractability of reproduction.

(1) Tractability of populations. Due to conspicuous nature of sessile pupal bags tightly attached onto host plants by larvae before pupation, abundance and demography of bagworms can be assessed in local populations by collecting cohorts of bags at repeated intervals from the onset to the end of the emergence cycle [22,24,25].
(2) Tractability of male and female emergence. Pupae of bagworms exhibit strong sexual dimorphism (♂: obtect pupa; ♀: barrel-shaped pupa; Figure 1 in [26], Figure 6 in [27]) and can be readily sexed by cutting each pupal bag open to examine its content (‘dissection’). Emergence of adult males is indicated by an empty pupal case (or parts thereof) hanging at the lower extremity of the bag; winged males actively seek sexually receptive females. Sessile neotenic females emerge by splitting the anterior segment of their pupal case to expel pheromone-impregnated setae in the lower section of the bag [28,29]. Dissemination of pheromone outside the bag is passive and to some extent independent of female behavior [30,31], thus calling female bagworms are deemed in ‘true’ permanent mating time-in phase when they are alive—with the caveat that attraction does not stop with physiological death of females [30,31].
(3) Tractability of female reproductive status. Female bagworms entirely reproduce within their pupal case inside their bag (attraction of males, copulation, oviposition). The presence of a live female within her split pupal case inside a bag with pheromone-impregnated seta is indicative of an actively calling female; each ‘candidate’ calling female was removed from her pupal case to determine presence/absence of eggs corresponding to either time-out mated females in the process oviposition and ‘true’ virgin calling females, respectively. Among time-out females, the presence/absence of eggs inside pupal cases is a tell-tale sign of mating status [32,33]. Mated females start laying eggs in their pupal case immediately after copulation and die shortly after oviposition is completed [26,34,35,36]. In contrast, the presence of a dead female in a bag with pheromone-impregnated setae but no eggs is indicative of an unmated female that died as lifelong virgin. In sexually reproducing bagworms, unmated females do not lay unfertile eggs in their pupal case, even when close to death [28,35,37].

The life history trajectory of the adult female bagworm is characterized by an obligatory pheromone calling stage (c) followed by one of two scenarios with associated individual reproductive output ř:

ř = Ƒ_s_    if  c → m (1)

ř = 0    if  c → v(2)

Both mated (m) and unmated (v: virgin) females are in time-out (Ŧ: dead females from ongoing generation); average fecundity in any species s is denoted as Ƒ_s_. For any given day i, the reproductive composition of adult populations (Ᾱ_i_) can be summed up as follows:

Ᾱ_i_ = n_♂i_ + n_♀i_ (c + m_Ŧ_ + v_Ŧ_) (3)

Considering that time-out females are *not* available to males for mating, the sex ratio of interest for any day i relates to the number of males per calling female (ᶘ_i_), which is assumed to be positively correlated with hypothetical (post sampling) mating probability of calling females (ℳ_ci_).

ᶘ_i_ = ♂_i_/c_i_ (4)

ℳ_ci_ = [m_ci_/(m_ci_ + v_ci_)] ᶘ_i,ȥ_ (5)

The variables m_ci_ and v_ci_ represent estimates of calling females that mate or die as lifelong virgins. Rules of thumb to quantify m_ci_/v_ci_ relative to ᶘ_i_ are detailed in Materials and Methods, including effects of adult longevity (ȥ).

In contrast with time-in females, mating probability among time-out females (ℳ_Ŧi_) can be empirically derived by dissecting pupal bags.

ℳ_Ŧi_ = m_Ŧi_/Ŧ_i_ (6)

Overall female mating success (ℳ_i_) can be approximated by combining Equations (5) and (6).

ℳ_i_ = (m_Ŧi_ + m_ci_)/♀_i_

Mating functions are bounded between ℳ_i_^−^ and ℳ_i_^+^, with assumptions that calling females either never or always mate:

ℳ_i_^−^ = m_Ŧi_/♀_i_

ℳ_i_^+^ = (m_Ŧi_ + c_i_)/♀_i_

The incidence and phenology of calling females (mating time-in) is explored with the Equations above in three species of bagworm [*Oiketicus kirbyi* Guilding, *Metisa plana* Walker, *Thyridopteryx ephemeraeformis* Haworth], factoring latitudinal clines in the latter species. The data set includes >2000 calling females with known sampling dates and locations that are described and analyzed for the first time (Table 1). The data are contextualized noting biological similarities/dissimilarities between species.

## 2. Materials and Methods

### 2.1. Life History of Three Bagworm Species

*Oiketicus kirbyi* and *Metisa plana* are defoliators of oil palm, *Elaeis guineensis* Jacquin, in Central–South America and Southeast Asia (Table 2). Major interspecific differences relate to the short development, small body size, and low fecundity of *M. plana* relative to *O. kirbyi* (Table 2): the progeny of one female *O. kirbyi* has the potential to defoliate leaf areas equivalent to the progeny of 600 female *M. plana* (Table 3 in [41]). Taxonomically, *M. plana* and *O. kirbyi* belong to the subfamilies Metisinae and Oiketicinae (Table 2), with male attraction in both subfamilies involving pheromone impregnated setae shed by females in their bag. The setae shed by emergent female *M. plana* are reddish in *M. plana* and bright yellow in *O. kirbyi* and *T. ephemeraeformis*. Upon emergence, virgin female Metisinae periodically protrude their head and thoracic segments from the lower section of the bag to further pheromone dissemination and mate attraction; a similar behavior has commonly been observed in old (near death) calling female Oiketicinae [42,43,44].

*Thyridopterix ephemeraeformis* (Oiketicinae subfamily) is native to temperate North America where it defoliates ornamental trees in urban–suburban areas, predominantly *Juniperus* and *Thuja* species [53,54]. Body size and fecundity of females is intermediate between *M. plana* and *O. kirbyi* (Table 2). Unlike the two other species, *T. ephemeraeformis* is univoltine with an obligatory overwintering egg stage [55].

### 2.2. Sampling Procedures

**Figure 1:**
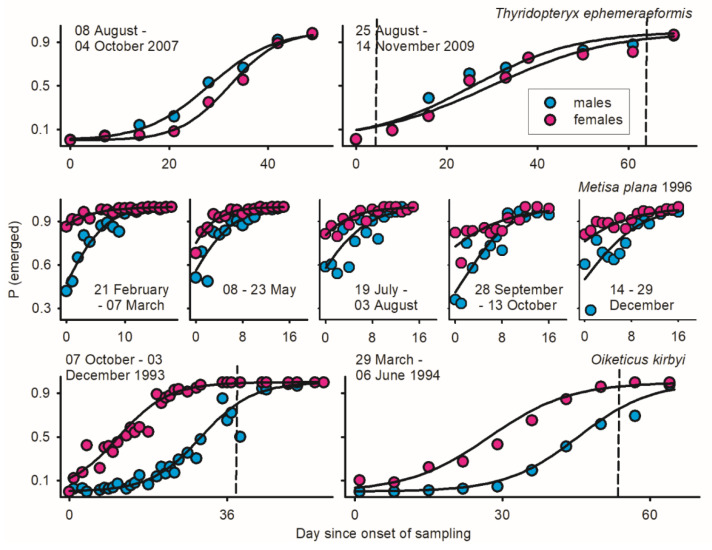
Patterns of adult emergence for three species of bagworms [adult/(pupa + adult)] using day since onset of sampling and zero intercept (day i at onset = 0) as time proxy in analysis. (1) *Oiketicus kirbyi* in plantation of oil palm in Costa Rica in 1993–1994 (Figure 2 in [38]). (2) *Metisa plana* in Malaysian plantation of oil palm during five consecutive generations in 1996 (G1 to G5) (Figure 5 in [39]). (3) *Thyridopteryx ephemeraeformis* on ornamental trees in Central Indiana in 2007 [25] and between North Tennessee–South Michigan in 2009 [22]; data in 2008 are not depicted due to short time series of abundance (14 days interval). Parameters of logistic regressions for males and females as provided in Table A1 in Appendix A. Dashed vertical lines represent time intervals with no calling female; these intervals were deleted from subsequent analyses.

Populations of *O. kirbyi* were sampled in Costa Rican oil palm plantations during two generations at Coto 50 and Coto 52, approximately 5 km apart (ca 8.62° N 82.98° W). In 1993, pupal bags were collected on all fronds of 170 young palms (3 to 4 m high) at near daily intervals between October 7 and December 3 [38]. The same procedure was used in 1994 by sampling three to nine fronds of 210 ‘old’ palms (4 to 5 m high) at weekly intervals between March 29 and June 6 [39]. Bagworms were sampled from the onset to the end of the emergence cycle (Figure 1). As in any data set below, pupal bags were all cut open to determine the abundance, sex ratio, and reproductive status of females. Early/late season intervals with no calling female (vertical dashed lines in Figure 1) are excluded from analysis.

Due to the short generation cycle of *M. plana* combined with sedentary (non-dispersing) larval progeny [41], bagworms from past generations tend to accumulate on old fronds of oil palm. Sampling was thus conducted on one young frond in the upper canopy (<2 months old) which only carried individual bagworms from the ongoing generation. The same palms located in a plantation near Sungei Merah in Peninsular Malaysia were sampled during five consecutive generations of *M. plana* in 1996. The period of sampling did not cover the entire emergence cycle, focusing instead on the second half of emergence with >50% adult emergence. A sexual segregation of the pupation site (females systematically pupate at higher locations than males: [38,50,51]) is often observed in bagworms, including *M. plana* [41]. Because only one frond in the upper canopy was sampled, the abundance of males relative to females was multiplied by a conservative factor of two (Table 2 in [41]).

Pupal bags of *T. ephemeraeformis* were collected in 2007 on infested trees located within a 50 km radius around Lafayette in Indiana (40°–40.5° N) at weekly intervals between August 18 and October 4 [25]. Data in 2008 included 119 sites sampled once between 3 and 16 October within latitudinal range 39.56°–41.28° N [40]. Data in 2009 included 71 sites (up to 9 sampling intervals between 2 September and 14 November) over a wide geographic range (38.39° to 41.74° N) including Tennessee, Kentucky, Indiana, and Michigan (Figure 2 in [22]).

### 2.3. Data Analysis

All statistical analyses were conducted with the SAS package version 9 (SAS Institute, Cary, NC, USA). Each data set (*O. kirbyi* in 1993 and 1994; five generations of *M. plana* in 1996; and *T. ephemeraeformis* in 2007–2009) was standardized using day i = 0 at the onset of female calling.

The response variable in Equation (4) (ᶘ_i_) is computed as the ratio of males per calling female using average values on a per day basis. Cumulative numbers of males/calling females up to day i (ñ_♂i_/Ʃ♂_i_, ñ_ci_/Ʃc_i_) are modelled with logistic regression.

*P* (ñ_♂i_) = e ^(ƍ+ƍi)^/[1 + {e ^(ƍ+ƍi)^}] (7)

*P* (ñ_ci_) = e ^(γ+γi)^/[1 + {e ^(γ+γi)^}] (8)

Parameters of logistic models (summarized in Table A1 and Table A2 of Appendix A) were used to infer number of emerged males and calling females between intervals to derive estimates of ᶘ_i_. Because male bagworms typically die within one day after emergence [12], number of live adult males for any day i is equivalent to daily variation in abundance (♂_i+1_ − ♂_i_). Female bagworms are long-lived relative to males, but no tool is available to ‘age’ feral calling females at the time of sampling. The issue is circumvented by assuming that calling females are ‘young’ (<one day old) at the time of sampling (ñ_ci_ = 0), with longevity (ȥ) set as either 1, 3, or 9 days. Under these conditions, ratios of males per calling female can be derived for any interval with the equations above.

ᶘ_(0,ȥ)_ = (Ʃñ_♂(ȥ)_ − ñ_♂i=0_)/ñ_i=0_ (9)

Estimates of ℳ_ci_ account for the relative abundance of males on any day i to derive mating probability of calling females (Equation (5)). Rules of thumb to infer the sex-ratio-dependent mating probability of calling females are as follows: ℳ_ci_ = 0 when ᶘ_(0,ȥ)_ < 0.5; ℳ_ci_ = 0.25 when 0.5 < ᶘ_(0,ȥ)_ < 1; ℳ_ci_ = 0.5 when 1 ᶘ_(0,ȥ)_ < 2; ℳ_ci_ = 0.75 when 2 < ᶘ_ȥ(0,ȥ),_ < 4; and ℳ_ci_ = 1.0 when ᶘ_ȥ(0,ȥ)_ > 4.

## 3. Results

### 3.1. General Trends

Proportion of live calling females (c/♀) was low in *M. plana* (5.0%), intermediate in *O. kirbyi* (12.3%), and high in *T. ephemeraeformis* (16.0%) (Table 1). Caution is warranted as to the interspecific rate of mating time-in due to systematically different sampling protocols in *O. kirbyi* and *M. plana*, as well as latitudinal clines in *T. ephemeraeformis* (see below).

**Figure 2:**
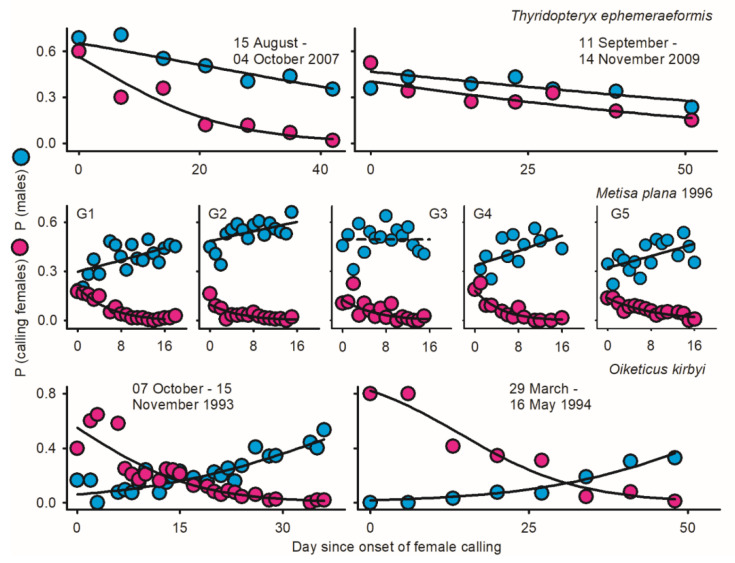
Temporal variation in proportions of males in adult populations [*P* (♂i/Āi), blue dots] and calling females [*P* (ci/♀i), pink dots] in three species of bagworms: *Oiketicus kirbyi* (1993–1994), *Metisa plana* during five consecutive generations in 1996 (G1 to G5), and *Thyridopteryx ephemeraeformis* in 2007 and 2009; data in 2008 are not depicted due to the short time series of abundance (14 days interval). Day since onset of female calling (set as day 0) was used as a time proxy in all analyses. Parameters of logistic regressions are as reported in Table A1 in Appendix A. Detailed sampling procedures and references are as shown in Figure 1.

**Figure 3:**
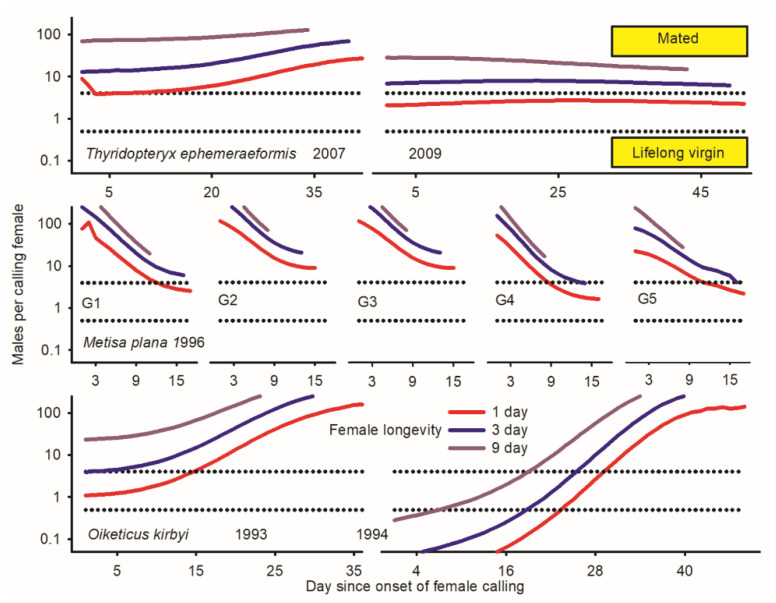
Temporal variation in ratios of males per calling females (ᶘ_(0,ȥ)_ in Equation (9)) in three species of bagworms: *Oiketicus kirbyi* (1993–1994), *Metisa plana* during five consecutive generations in 1996 (G1 to G5), and *Thyridopteryx ephemeraeformis* in 2007 and 2009; data in 2008 are not depicted due to the short time series of abundance (14 days interval). Proportion of calling females that mate (ℳ_ci_, as in Equation (5)) was derived using ratios of males per calling females with variable longevity (ȥ = 1, 3, 9 days) as represented by horizontal dotted lines: (1) ℳ_ci(ᶘ)_ = 0 when ᶘ_(0,ȥ)_ < 0.5; (2) ℳ_ci(ᶘ)_ = 0.25 when 0.5 < ᶘ_(0,ȥ)_ < 1.0; (3) ℳ_ci(ᶘ)_ = 0.50 when 1.0 < ᶘ_(0,ȥ)_ < 2.0; (4) ℳ_ci(ᶘ)_ = 0.75 when 2.0 < ᶘ_(0,ȥ)_ < 4.0; and (5) ℳ_ci(ᶘ)_ = 1 when ᶘ_(0,ȥ)_ > 4.0. Day since onset of female calling (set as day 0) was used as a time proxy in all analyses. Parameters of logistic regressions are as reported in Table A1 in Appendix A. Detailed sampling procedures and references are as shown in Figure 1.

**Figure 4:**
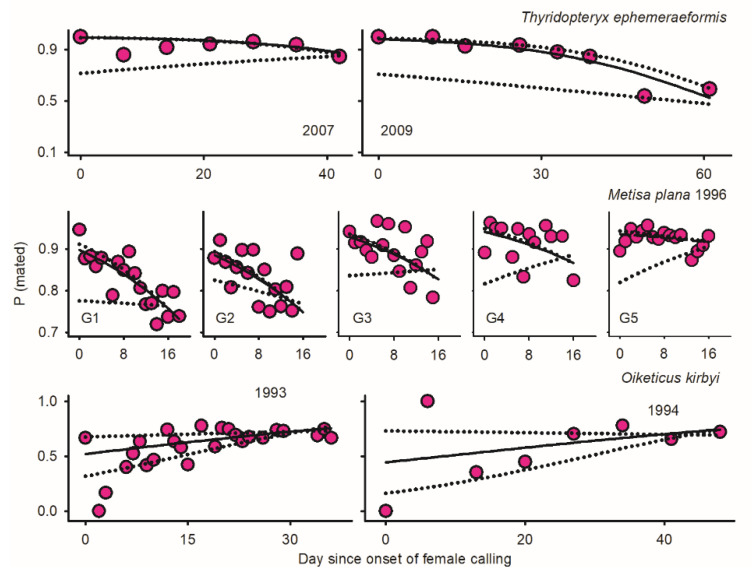
Temporal variation of mating probability among time-out females (Equation (6)) in three species of bagworms: *Oiketicus kirbyi* (1993–1994), *Metisa plana* during five consecutive generations in 1996 (G1 to G5), and *Thyridopteryx ephemeraeformis* in 2007 and 2009; data in 2008 are not depicted due to the short time series of abundance (14 days interval). The solid lines represent the proportion of time-out females that mated as adults; dotted lines represent low- and high-function boundaries assuming that either calling females never mate or always mate (Equations (8) and (9)). Day since onset of female calling (set as day 0) was used as a time proxy in all analyses. Parameters of logistic regressions are reported in Table A1 in Appendix A. Detailed sampling procedures and references are as shown in Figure 1.

Protogyny in *O. kirbyi* and *M. plana* versus protandry in *T. ephemeraeformis* (Figure 1) predictably lead to decline/increment in relative abundance of males over time [*P* (♂_i_/Ā_i_)] (Figure 2). The proportion of calling females significantly declined over time for all data sets (Figure 2)—as expected in populations with synchronous (non-overlapping) reproductive generations (see Discussion). With the exception of *O. kirbyi* in 1994, all calling females are expected to mate when adult longevity (ȥ) is ≥3 days (Figure 3). Mating success of time-out females declined over time for *M. plana* and *T. ephemeraeformis*, as opposed to an increase in mating probability over time in *O. kirbyi* (Figure 4).

### 3.2. Oiketicus kirbyi

Adult females outnumbered males by a factor of ca 3.0 in 1993 and 1994 (Table 1). The extent of protogyny (interval between median date of female and male emergence) exceeded two weeks each year (Figure 1). More than two thirds of females were sampled as live calling at the onset of emergence, in part due to the low abundance of males; the proportion of calling females remained above 25% for about two weeks (Figure 3). Mating probability of time-out females increased over time (Figure 4) and ranged between 67.4 and 68.3% on a per year basis (Table 1).

In 1993, calling females were all expected to mate irrespective of time if they lived at least three days; in contrast, mating probability of short-lived females (one day) increased from 50 to 100% during the first 15 days of sampling and remained above 100% thereafter (Figure 3). Overall (season-long) mating probability of calling females increases with longevity from 77.1% (103.3/134) for a one-day lifespan to 100% for a lifespan > 3 days (Figure 3)

Under assumptions that calling females survive either one or three days in 1994, null (zero) mating success is expected during the first 24/18 days of sampling. Overall mating probability of calling females increases with longevity from 38.3% (34.5/90) for a one-day lifespan to 66.1% for a three-day lifespan and 84.4% for a nine-day lifespan (Figure 3).

### 3.3. Metisa plana

Depending upon generation G, females outnumbered males (G1, G4, and G5) or were outnumbered by males (G2 and G3); overall, the proportion of males averaged 45.2% of individuals (6630 ♂, 8046 ♀) (Table 1). Due to protogyny, the proportion of males relative to females significantly increased over time during four of five generations (Figure 2). The extent of protogyny, defined as the interval corresponding to 85% adult emergence for males and females, averaged 4.7 days on a per generation basis (Figure 1). The proportion of live calling virgins declined from *ca* 20% at the onset of sampling to <2% at the end of reproductive season, averaging 5.1% on a generation basis (Figure 2). The mating probability of time-out females declined over time (Figure 4) and ranged between 80.7 and 92.7% for different generations (Table 1).

Ratios of males per calling female generally exceeded 4:1 (Figure 3), implying that most calling females mated during their lifetime [estimates of 100% for lifespan > 3 days in all generations]. Even when assuming short (one day) longevity, more than 96% of calling females were expected to mate during each generation (Figure 3).

### 3.4. Oiketicus kirbyi Versus Metisa plana

Sampling protocols for *O. kirbyi* and *M. plana* fundamentally differed. Adults were sampled during the entire emergence cycle for *O. kirbyi*, as opposed to the late portion of emergence in *M. plana* (>50% emerged males and 85% emerged females) (Figure 1 and Figure 2). Comparison between species was standardized in *O. kirbyi* by using only late season estimates corresponding to >85% female emergence (21 and 42 days after onset of sampling in 1993 and 1994, respectively; Figure 1). Under this scenario, female *M. plana* were more than twice as likely to be sampled as live calling females than in *O. kirbyi* [*M. plana*: 405 of 8046 females, or 5.1%; *O. kirbyi*: 47 of 2216 females, or 2.1%].

### 3.5. Thyridopteryx ephemeraeformis in 2007–2009: Data Pooled by Year

Males were outnumbered by females each year, representing 35.1 to 43.2% of adults (Table 1). The extent of protandry (interval between median date of male and female emergence) lasted 3.8 days in 2007, <1 day in 2008, and 3.3 days in 2009 (Figure 1). On a per site basis, however, all patterns of emergence were observed: early, synchronous, and late emergence of males relative to females [56].

The proportions of males and calling females declined over time each year (Figure 2). The proportion of calling females was low in 2007 (6.6%), intermediate in 2008 (16.4%), and high in 2009 (23.7%) (Table 1). Mating probability of time-out females was high in 2007 (85.3%) and 2008 (87.5%), but low in 2009 (72.1%) (Table 1). Based on relative abundance of males, all calling females in 2007 are expected to mate (Figure 3). When data were pooled across latitudes in 2009, mating success of calling females with a one-day lifespan varied within a narrow range around 75% during the entire reproductive season; all females were expected to mate when longevity exceeded 3 days (Figure 3).

### 3.6. Thyridopteryx ephemeraeformis in 2007

Trends observed in 2007 *cannot* be extrapolated to any other year because bagworms were collected on a narrow geographic range (50 km radius around Lafayette in Indiana), as opposed to a large spatial range in 2008–2009 (including populations at southern and northern latitude that exhibit a very low/high incidence of female lifelong virginity [22,40]). As an example, data collected in Indiana at one site 120 km north of Lafayette (Hamlet: 41.28° N, 86.58° W) in late October 2007 at the end of the reproductive season (no calling females, only time-out females) revealed a low female mating probability (1079 of 1854 individuals, or 58.2% [57]) compared to 85.3% for mated females sampled between 40.0 and 40.5° N (Table 1).

### 3.7. Thyridopteryx ephemeraeformis in 2008

Based on the observation at Hamlet in 2007, a two-week haphazard experiment was conducted in 2008 including 118 sites sampled *once* between 3 and 16 October within a latitudinal range 39.56–41.28° N [40]. Logistic regression including two independent variables [day, latitude] revealed the following trends (Table 3). (1) The relative abundance of males increased with time and latitude. (2) Time-independent proportion of calling females increased with latitude. (3) Time-independent mating probability among time-out females declined with latitude (Table 4).

### 3.8. Thyridopteryx ephemeraeformis in 2009

**Figure 5:**
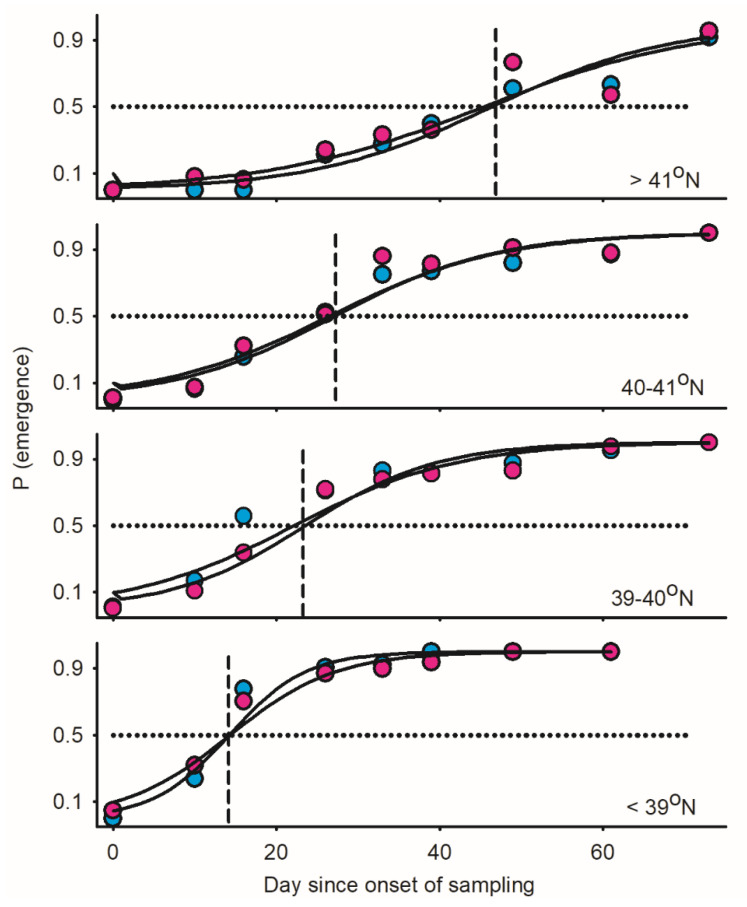
Patterns of adult emergence [adult/(pupa + adult); blue and red dots for males and females] in *Thyridopteryx ephemeraeformis* populations sampled on ornamental trees in 2009 in the Midwest United States between North Tennessee and South Michigan (38.39–41.74° N (Figure 2 in [22]). Day since onset of emergence (day i = 0 at onset) was used as a proxy of time. Data were divided into four latitudinal bands for analysis. Parameters of logistic regression models are listed in Table A2 in Appendix A. Dotted and dashed lines represent dates corresponding to 50% adult emergence.

**Figure 6:**
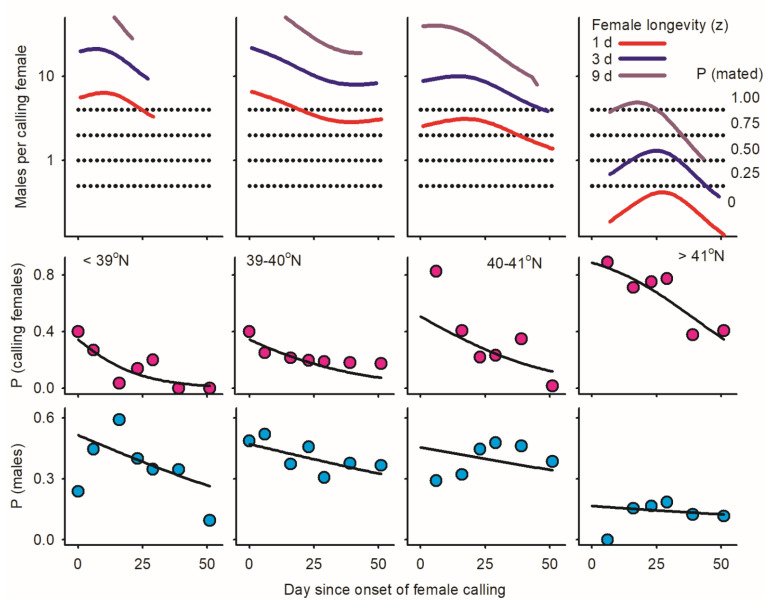
Patterns of adult reproduction in *Thyridopteryx ephemeraeformis* populations sampled on ornamental trees in 2009 in the Midwest United States between North Tennessee and South Michigan (38.39–41.74° N; Figure 2 in [22]). Day since onset of sampling was used as a proxy of time, and data were divided into four latitudinal bands for analysis. Lower plots: proportion of males in adult populations [*P* (♂i/Āi), blue dots]. Middle plots: proportions of calling females among adults [*P* (ci/♀i), pink dots]. Upper plots: proportion of calling females that mate (ℳ_ci_, as in Equation (5)) as derived using ratios of males per calling females with variable longevity (ȥ = 1, 3, 9 days) as represented by horizontal dotted lines: (1) ℳ_ci(ᶘ)_ = 0 when ᶘ_(0,ȥ)_ < 0.5; (2) ℳ_ci(ᶘ)_ = 0.25 when 0.5 < ᶘ_(0,ȥ)_ < 1.0; (3) ℳ_ci(ᶘ)_ = 0.50 when 1.0 < ᶘ_(0,ȥ)_ < 2.0; (4) ℳ_ci(ᶘ)_ = 0.75 when 2.0 < ᶘ_(0,ȥ)_ < 4.0; and (5) ℳ_ci(ᶘ)_ = 1 when ᶘ_(0,ȥ)_ > 4.0. Parameters of logistic regression models are listed in Table A2 in Appendix A.

Data were divided into four latitudinal bands prior to analysis [<39°, 39–40° N, 40–41° N, >41° N]. Logistic regression models simultaneously factoring in day/sex/latitude revealed similar patterns of emergence for males and females (Wald ꭓ^2^ = 2.43, *p* = 0.1191) with steady increases in emergence over time (Wald ꭓ^2^ = 1563.71, 0.0001) and delayed emergence with increasing latitude (30 days difference between southernmost and northernmost latitudinal bands: Wald ꭓ^2^ = 485.48, *p* < 0.0001) (Figure 5). Both the proportions of males and calling females in adult populations decreased over time for all latitudinal bands (Figure 6). Most strikingly, proportion of calling females relative to time-out females increased from south to north; the same trend was observed, to a lower extent, for the proportion of males (Figure 6). When the trends above are combined, calling females are expected to mate at the following rates relative to latitude.

**Figure 7:**
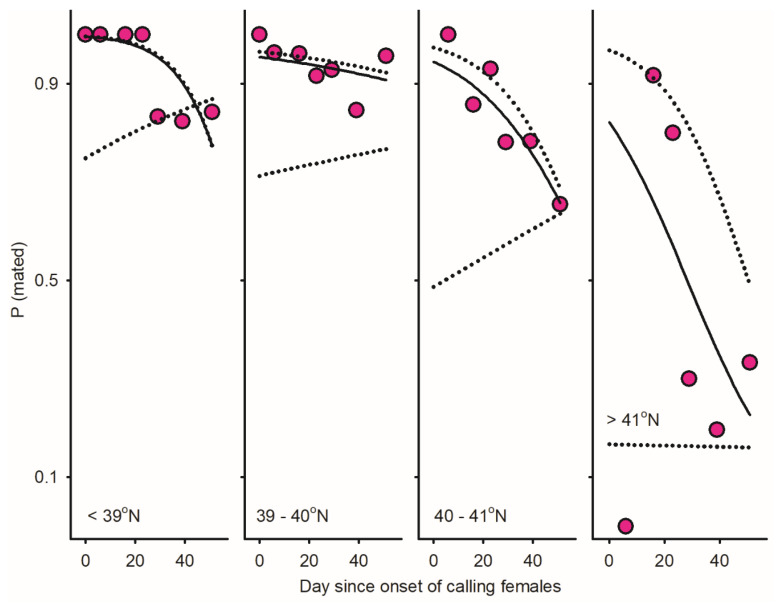
Temporal variation in mating probability among time-out females (Equation (6)) in *Thyridopteryx ephemeraeformis* populations sampled on ornamental trees in 2009 in the Midwest United States between North Tennessee and South Michigan (38.39–41.74° N; Figure 2 in [22]). Day since onset of sampling was used as a proxy of time, and data were divided into four latitudinal bands for analysis. The solid lines represent the observed proportion of mated females; dotted lines represent low- and high-function boundaries assuming that either calling females never mate or always mate (Equations (8) and (9)). Parameters of logistic regression models are listed in Table A2 in Appendix A.

(1) Assuming that longevity of females is equal to or exceeds 3 days, ratios of males per calling female (ᶘ) exceeded 4.00 for any day in the three southernmost latitudinal bands (<41° N), implying that all calling females mated as adults.
(2) Assuming that females live for one day or less, the probability that calling females mate as adults declines with latitude for the three southernmost bands [92.4% at <39° N, 86.7% between 39 and 40° N; and 70.1% between 40 and 41° N].
(3) Mating probability was lowest at latitudes >41° N (N = 173 calling females) and increased with female longevity (ȥ) from 0% when ȥ = 1, 34.2% when ȥ = 3, and 73.7% when ȥ = 9 (Figure 6 and Figure 7).

## 4. Discussion

In organisms with obligatory sexual reproduction, females are born virgin and need to access male sperm to produce viable offspring. This problem is simplified in holometabolous insects with complete metamorphosis because food acquisition and sexual activities are segregated into separate life history phases (transition between larval, pupal, and adult stages) [58,59,60]. Sessile female bagworms (Lepidoptera: Psychidae) reproduce within a self-constructed bag, thus providing a model system to parameterize the incidence of live ‘pheromone-calling’ females relative to time-out females. The low mating success of calling females is associated with extreme protogyny (early season male shortage; *O. kirbyi* in 1994) or late adult emergence in populations at the edge of the distribution range (*T. ephemeraeformis* at latitudes >41° N in 2019). Parameter estimates related to the reproductive status of females and sex ratio among adults, as derived here for Psychidae, could in theory be applied to three other taxonomic groups with common female monandry/flightlessness/neoteny: fireflies (Coleoptera: Lampyridae), twisted wings parasites (Strepsiptera), and scale insects (Hemiptera: Pseudococcidae) [12,17,61,62,63,64].

The definition of female mating time-in as developed in this study is nearly equivalent to live virginity, notwithstanding the passive emission of pheromone in bagworms [30,31]. Surveys of feral Lepidoptera captured with sweep nets or light traps often include punctual female mating success at the time of sampling (presence/absence of spermatophore or mating plug in the reproductive tract of females [10,65,66]. While these data are similar analytically to the incidence of mating time-in/time-out in female bagworms, future mating probability of live virgins is deemed unknowable in the absence of repeated measurements of abundance over time (including males, virgin females, and mated females). Data collected in female spruce budworms, *Choristoneura fumiferana* Clemens (Lepidoptera: Tortricidae), provide a system to link punctual virginity (based on proportion of mated females along age classes: young females foraging on host trees and mid-aged in-flight females captured at traps) and lifelong virginity (dead females collected on drop trays) [67,68,69,70]. 

Synchronous larval development in most bagworm species (including *O. kirbyi*, *M. plana*, *T. ephemeraeformis;* Table 1) leads to discrete (non-overlapping) reproductive generations, with males and females all emerging within a predetermined period. Considering monotonic trends of emergence patterns in discrete generations, the incidence of live calling females was higher at the onset of emergence (‘young’ females) than in the late season (when samples include mostly time-out females that previously mated or died as lifelong virgins). This mechanistic effect may be magnified in protogynous populations (early season female-biased sex ratio) relative to protandrous populations (early season male-biased sex ratio)—although similar trends were observed for protogynous *O. kirbyi*/*M. plana* and protandrous *T. ephemeraeformis* in this study. A moderate level of protandry is expected to increase the mating success of females/reduce the probability that live females are sampled while pheromone calling [56]. As hypothesized, negative effects of reproductive isolation on the mating success of protogynous female bagworms are buffered by a long lifespan [71,72]. From an applied perspective, populations of adult female *T. ephemeraeformis* can be managed with mating disruption using synthetic sex pheromone [28,29]. As in this study, the efficacy of mating disruption was assessed by recording numbers of live pheromone-calling ♀ (*c*), time-out mated ♀ (m_Ŧ_), and lifelong virgin ♀ (v_Ŧ_) females. Trees treated with pheromone consistently suppressed mating success among time-out females (40 of 310 females, or 12.9%) relative to control trees (254 of 308 females, 82.5%) (Table 1 in [28]). Re-invasion of treated plots by mated gravid females generally limits the feasibility of pheromone-based management in moths [73,74,75,76]; the issue is irrelevant in bagworms with sessile (non-dispersing) females.

Management of adult female *M. plana* in oil palm plantations may be achieved by mass trapping males with sticky traps each baited with two live calling females (thirty 60 × 30 cm traps/0.7 ha) [77]. Efficacy of mass trapping was assessed by recording the number of bags with eggs relative to the total number of female bags dissected (*m_Ŧ_*/♀); mass trapping reduced ‘mating probability’ of females from 35.7% (322 of 903 bags) to 20.3% (172 of 848 bags) (Table 1 in [77]). In addition to *c*, *m_Ŧ_*, and *v*_Ŧ_, the denominator may (or may not) have included female pupae (dead or alive) and females from past generations. More critically, the mass trapping experimental design may be fatally flawed as the control plots did not include sticky traps without calling females. It is conceivable that males were attracted to the large white surface of sticky traps per se, especially considering the large surface involved (7.7 m^2^/ha) (Figure 1 in [77]).

In principle, implementation of pheromone-based mating disruption/mass trapping requires a phenological model of adult emergence or monitoring tools to infer timing of emergence [78,79,80]. Other than the data provided here (Table 1), however, few studies report the phenology of bagworm reproduction in field conditions [81,82,83]. The paucity of data is surprising considering the ease of such measurements (relative to other insects) and the severe pest status of bagworms worldwide ([84], Table 4 in [85], Table 1 in [86], Table 1 in [87], Figure 2 in [88]).

## Figures and Tables

**Table 1:** Number of adult bagworms (♂, ♀) sampled in Costa Rican and Malaysian plantations of oil palm (*Ok*: *Oiketicus kirbyi* and *Mp*: *Metisa plana*) and on ornamental host trees in Midwest United States (*Te*: *Thyridopteryx ephemeraeformis*). Females were classified as in either mating time-in (c: calling female) or time-out (m, v = mated or virgin females). References as follows for *Ok* in 1993 [38] and 1994 (published herein for the first time), for *Mp* in 1996 [39], and for *Te* in 2007–2009 [22,25,40].

Species	Year	Gen	♂	♀	c	m	v	*P* (♂)	*P* (c)	*P* (m)
*Ok*	1993	1	438	1190	134	712	344	0.269	0.113	0.674
	1994	2	188	632	90	370	172	0.229	0.142	0.683
	Cumulative		626	1822	224	1082	516	0.256	0.123	0.677
*Mp*	1996	1	2056	3110	152	2388	570	0.398	0.049	0.807
		2	2000	1675	57	1339	279	0.544	0.034	0.828
		3	858	790	39	666	85	0.521	0.049	0.887
		4	710	916	50	783	83	0.437	0.055	0.904
		5	1006	1555	107	1342	106	0.393	0.069	0.927
	Cumulative		6630	8046	405	6518	1123	0.451	0.050	0.853
*Te*	2007	1	1342	1777	118	1478	181	0.433	0.066	0.853
	2008	2	3148	5306	851	3847	548	0.372	0.179	0.875
	2009	3	1096	2033	492	1112	429	0.350	0.242	0.722
	Cumulative		5586	9116	1461	6437	1158	0.380	0.160	0.848

**Table 2:** Summary of life history traits for three bagworm species (Lepidoptera: Psychidae). References for *Oiketicus kirbyi*: [26,34,38,41,45,46]; for *Metisa plana*: [36,39,41,47,48]; and for *Thyridopteryx ephemeraeformis*: [25,33,34,49,50,51,52].

	Oiketicinae		Metisinae
Species	*O. kirbyi*	*T. ephemeraeformis*	*M. plana*
Continent	Central/South America	North America	Southeast Asia
Climate	Tropical–Subtropical	Continental/Temperate	Tropical/Subtropical
Host plants	Oil palm	Juniper/Red cedar	Oil palm
Landscape	Commercial plantation	Urban/Suburban	Commercial plantation
Voltinism	1–2 generations/year	1 generation/year	3–5 generations/year
♀ length (mm)	34	24	12
Fecundity	5000	1000	130
Discrete generations	Yes	Yes	Yes

**Table 3:** Number of adult *Thyridopteryx ephemeraeformis* (♂, ♀) sampled and on ornamental host trees in Midwest United States. Females were classified as in either mating time-in (c: calling female) or time-out (m, v = mated or virgin females). References are as listed in Table 1 and Table 2.

Year	Latitude	♂	♀	c	m	v	*P* (♂)	*P* (c)	*P* (m)
2008	<40° N	704	1 123	83	943	97	0.385	0.074	0.881
	40–41° N	2 142	3 769	647	2 737	385	0.362	0.172	0.877
	>41° N	302	414	141	207	66	0.422	0.341	0.758
2009	<39° N	108	158	22	128	8	0.406	0.139	0.941
	39–40° N	549	843	145	604	94	0.394	0.172	0.865
	40–41° N	364	565	149	284	132	0.392	0.264	0.683
	>41° N	52	329	176	53	100	0.136	0.535	0.346

**Table 4:** Effects of latitude/day of sampling (i/j) on three response variables (proportion of males among adults, proportion of calling females among adult females, and proportion of mated females among time-out females) for *Thyridopteryx ephemeraeformis* adults sampled in 2008; statistical effects of i/j were derived with logistic regression models. Unless otherwise specified (ns superscript), parameters were all statistically significant (*p* < 0.01). Day since onset of sampling (set as day 0) was used as a time proxy in all analyses.

Variable	Intercept	∆_i_ Latitude	∆_j_ Day
*P* (♂_i,j_/Ā_ij_)	−8.42 ± 2.30	0.193 ± 0.056	0.0159 ± 0.0061
*P* (c_i,j_/♀_ij_)	−62.28 ± 3.88	1.500 ± 0.095	−0.0018 ± 0.0106 ^ns^
*P* (m_i,j_/Ŧ_i,j_)	35.08 ± 4.71	−0.823 ± 0.116	0.0116 ± 0.0120 ^ns^

## Data Availability

The original contributions presented in this study are included in the article. Further inquiries can be directed to the corresponding author.

## Appendix A

Note to Figure 1. Note [The proportion of emergence could in theory be used to infer variation in number of adults (A) relative to pupae (P) combined with sex-specific survival during the pupal stage (S) for males [*P* (♂i) = s♂ *P* (♂a/{♂p + ♂a}i)] and females [*P* (♀i) = s♀ *P* (♀a/{♀p + ♀a}i)]. Estimates of s♂ and s♀ are difficult to infer among species, even more so when they vary over time. For those reasons, individuals in the pupal stage were discarded from analysis. Functions of emergence above assume that s is equal to 1 (100% PUPAL survival) for both males and females].

**Table A1:** Parameters of logistic regression models describing emergence patterns of males and females (Figure 1), proportions of males and calling females in adult populations (Figure 2), mating probability of time-out females (Equation (6) in text), and cumulative abundance of adult males and calling females (Equations (8) and (9)) in text). Data sources for the three bagworm species under investigation (*Oiketicus kirbyi*: *Ok*; *Metisa plana*: *Mp*; *Thyridopteryx ephemeraeformis*: *Te*) as summarized in Table 1 and Table 2. Unless specified (ns), all logistic regressions were statistically significant.

Species		Adult Emergence				*P* (Males)		*P* (Calling)		*P* (Mated)		Cumulative Abundance			
		*P* (Ā_♂__i_/*N_♂__i_*)		*P* (*Ā_♀__i_*/*N_♀__i_*)		*P* (*♂_i_*/*Ᾱ_i_*)		*P* (*c_i_*/*♀_i_*)		*P* (*m_i_*/*Ŧ_i_*)		*P* (Ʃ*ñ_♂__i_/*Ʃ*♂_i_*)		*P* (Ʃ*ñ_ci_*/Ʃ*c_i_*)	
		β_0_	β_1_	α_0_	α_1_	Ω_0_	Ω_1_	θ_0_	θ_1_	δ_0_	δ_1_	ƍ_0_	ƍ_1_	γ_0_	γ_1_
*Ok*	1993	−5.17	0.17	−1.97	0.18	−2.74	0.07	0.2	−0.13	0.08	0.03	−5.4	0.12	−2.88	0.22
	1994	−6.2	0.14	−3.33	0.12	−3.91	0.07	1.54	−0.11	−0.22	0.03	−9.65	0.25	−3.32	0.16
*Mp*	G1	−0.61	0.31	1.68	0.25	−0.85	0.04	1.37	−0.2	2.17	−0.07	−3.56	0.33	−0.86	0.27
	G2	−0.23	0.27	0.82	0.38	−0.05	0.03	−2.21	−0.19	2.04	−0.06	−2.98	0.39	−1.19	0.36
	G3	−0.2	0.25	1.11	0.28	0.11	0.00 ^ns^	−1.93	−0.18	2.56	−0.06	−2.39	0.35	−1.07	0.38
	G4	−0.84	0.25	0.7	0.16	0.38	0.05	−1.51	−0.24	2.76	−0.06	−2.8	0.36	−0.31	0.34
	G5	−0.54	0.22	1.17	0.17	−0.74	0.04	−1.84	−0.13	2.67	−0.02	−2.45	0.32	−1.44	0.36
*Te*	2007	−3.24	0.15	−5.08	0.2	0.64	−0.03	0.26	−0.09	4.88	−0.07	−5.41	0.17	−4.2	0.17
	2009	−2.47	0.09	−2.44	0.08	−0.12	−0.02	−0.39	−0.02	3.23	−0.06	−2.71	0.12	−2.39	0.11

**Table A2:** Parameters of logistic regression models describing demography of *Thyridopteryx ephemeraeformis* along four latitudinal bands in 2009: emergence patterns of males and females (Figure 5); proportions of males and calling females in adult populations (Figure 6); mating probability of time-out females (Figure 7); and cumulative abundance of adult males and calling females (Equations (8) and (9) in text). Data sources are as summarized in Table 1 and Table 2. Unless specified (ns), all logistic regressions were statistically significant.

Latitude	Adult Emergence				*P* (Males)		*P* (Calling)		*P* (Mated)		Cumulative Abundance			
	*P* (Ā_♂__i_/*N**_♂_**_i_*)		*P* (*Ā**_♀_**_i_*/*N**_♀_**_i_*)		*P* (*♂**_i_*/*Ᾱ_i_*)		*P* (*c_i_*/*♀**_i_*)		*P* (*m_i_*/*Ŧ_i_*)		*P* (Ʃ*ñ**_♂_**_i_**/*Ʃ*♂**_i_*)		*P* (Ʃ*ñ_ci_*/Ʃ*c_i_*)	
	β_0_	β_1_	α_0_	α_1_	Ω_0_	Ω_1_	θ_0_	θ_1_	δ_0_	δ_1_	ƍ_0_	ƍ_1_	γ_0_	γ_1_
<39° N	−3.04	0.21	−2.25	0.16	0.11	−0.02	0.4	−0.07	6.25	−0.08	−2.77	0.19	−1.63	0.11
39–40° N	−2.22	0.1	−2.91	0.12	−0.07	−0.01	−0.54	−0.03	4.41	−0.06	−2.53	0.11	−1.82	0.1
40–41° N	−2.93	0.1	−2.68	0.1	−0.14	−0.01	−0.05	−0.04	2.22	−0.03	−3.3	0.14	−1.61	0.1
41–42° N	−4.19	0.09	−3.43	0.07	−1.51	−0.01 ^ns^	2.93	−0.06	2.07	−0.05	−5.32	0.19	−2.71	0.12
